# Impact of Multiple Cardiovascular Risk Factors on Carotid Intima-Media Thickness and Elasticity

**DOI:** 10.1371/journal.pone.0067809

**Published:** 2013-07-02

**Authors:** Lili Niu, Yanling Zhang, Ming Qian, Long Meng, Yang Xiao, Yuanyuan Wang, Xin Liu, Rongqin Zheng, Hairong Zheng

**Affiliations:** 1 Paul C. Lauterbur Research Center for Biomedical Imaging, Institute of Biomedical and Health Engineering, Shenzhen Institutes of Advanced Technology, Chinese Academy of Sciences, Shenzhen, China; 2 Department of Ultrasound, Third affiliated hospital, Sun Yat-sen University, Guangzhou, China; 3 Department of Electronic Engineering, Fudan University, Shanghai, China; Scientific Directorate, Bambino Hospital, Italy

## Abstract

**Background:**

Carotid intima-media thickness (IMT) and elasticity have been shown to be independent predictors of cardiovascular disease (CVD). Cardiovascular risk factors (CVRFs) includes hypertension, dyslipidemia, diabetes, overweight and smoking. The objective was to investigate whether the clustering of three or more components of CVRFs has a greater impact on carotid IMT and elasticity than individual components of CVRFs.

**Methods:**

One hundred and seventy-three participants without clinical CVD were classified as the multiple CVRFs patients with three or more CVRFs (n  = 55) and control group with two or less CVRFs (n  = 118). Carotid IMT and elastic modulus were measured by B-mode ultrasound and vessel texture matching method (VTMM), respectively.

**Results:**

The multiple CVRFs conferred a disproportionate increase in carotid IMT (43%, p<0.0001) and elastic modulus (60%, p<0.0001), compared with control group. Multiple regression models, which included age, gender, as well as each individual component of CVRFs as continuous variables, showed that multiple CVRFs was an independent determinant of both IMT (p  = 0.042) and elasticity (p  = 0.008). In the analysis of variance adjusted with age, subjects with single, double, and multiple CVRFs, increased by 8.1%, 42.2%, and 66% for IMT, 54.6%, 94.3%, and 125.2% for elastic modulus, respectively, compared to subjects without CVRFs.

**Conclusions:**

The clustering of multiple CVRFs has a greater impact on carotid IMT and elasticity than individual components of CVRFs. This suggests that the components of CVRFs interact to synergistically impact carotid IMT and elasticity.

## Introduction

Cardiovascular disease (CVD) is a leading cause of death worldwide. Alterations in vascular structure and function, including increased wall thickness, as indexed by intima-media thickness (IMT) and decreased arterial wall elasticity, are increasingly recognized as significant independent predictors of adverse cardiovascular outcomes [1]–[5].

The multiple cardiovascular risk factors (CVRFs) are defined as the clustering of three or more of the CVRFs in an individual, including hypertension, dyslipidemia, diabetes, overweight and smoking. The independent association between carotid IMT or elasticity and the individual components of CVRFs has previously been reported [6]–[11]. Although the risks for CVD associated with individual CVRFs have been previously examined [12]–[16], less information exists concerning the role of multiple CVRFs.

Therefore, a cross-sectional study was undertaken to investigate the relationship between multiple CVRFs and carotid structure (thickness) and function (elasticity). We aimed to assess whether the clustering of multiple components of CVRFs has a greater impact on carotid IMT and elasticity than individual components of CVRFs.

## Methods

### Subjects

Our study population consisted of 196 subjects (47 healthy volunteers and 149 patients with CVRFs) from the third affiliated hospital of Sun Yat-sen University were enrolled from October 2011 to August 2012. Twenty-three participants with coronary heart disease were excluded. Subjects (80 men and 93 women; age 17 to 79 years) free of clinically overt CVD were classified as the multiple CVRFs patients with three or more CVRFs (n  = 55) and control group with two or less CVRFs (n  = 118). The study protocol was approved by the Institutional Review Board of the third affiliated hospital of Sun Yat-sen university, and written informed consent was obtained from participants or on the behalf of minors/children participants from their next of kin, caretakers or guardians. Information regarding age, gender, blood pressure (BP), body mass index (BMI), history of smoking, total, low-density lipoprotein (LDL) and high-density lipoprotein (HDL) cholesterol, triglyceride, fasting plasma glucose, and medication use was available to all participants.

### Definition of Risk Factors

The criteria used were guided by Adult Treatment Panel III and the World Health Organization [17], [18]. Hypertension was defined as resting systolic blood pressure (SBP) ≥140mmHg and/or diastolic blood pressure (DBP) ≥90mmHg and/or the use of antihypertensive drugs. Dyslipidemia was defined using lipid-lowering drugs or having one or more of the following: total cholesterol ≥5.2mmol/L, LDL cholesterol ≥3.4mmol/L, HDL cholesterol ≤1.0mmol/L, or triglyceride ≥1.70mmol/L. Diabetes was defined as fasting plasma glucose ≥7.0mmol/L or the use of antidiabetic medication. Overweight was defined as a BMI ≥25.0kg/m^2^. Smoking status was ascertained by a questionnaire that classified each subject as a non-smoker, former smoker, or current smoker. For the purpose of the present study, “ever-smoker” status (former or current) was used.

### Carotid Ultrasonography

The study was performed by the same operator using an Esaote MyLab 90 ultrasound Platform (Esaote Medical Systems, Rome, Italy) equipped with a 4∼13 MHz linear-array transducer (LA523). Subjects lay in the supine position in a quiet room. The left common carotid artery (CCA) was examined with the head tilted slightly upward in the mid-line position. The transducer was manipulated so that the near and far walls of the CCA were parallel to the transducer footprint, and the lumen diameter was maximized in the longitudinal plane. A region 1∼2cm proximal to the carotid bifurcation was imaged, and the IMT of the far wall was evaluated as the distance between the lumen-intima interface and the media-adventitia interface. The IMT was measured on the frozen frame of a suitable longitudinal image, with the image magnified to achieve a much higher spatial resolution.

Elastic modulus of the CCA was evaluated by the vessel texture matching method that has been described and validated by Niu et al. [19]:
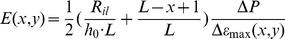
(1)where (*x*, *y*) correspond to the coordinates of a pixel in the image plane; *L*, *h*_0_, *R_il_*, Δ*ε*_max_ and Δ*P* are the number of layers, thickness of each layer, the inner radius of the *l*-th layer, the maximum strain of each layer during one cardiac cycle, and PP measured at the brachial artery, respectively.

### Statistical Analysis

All analyses were performed using software from Minitab, Inc (version 16, State College, PA). Data are presented as the mean value ± standard deviation (SD), unless otherwise specified. Differences in mean values for each of the measured variables in subjects with and without multiple CVRFs were compared by the *t* test for continuous variables and by the chi-square test for categorical variables. Pearson correlation coefficient was used for univariate analysis. A comparison of different age quartiles was made by analysis of variance (ANOVA). To evaluate the independent determinants of IMT and elasticity, multiple regression models were constructed, which included age, gender and each individual’s CVRFs as independent variables. Stepwise regression analysis was used to calculate the contribution of the significant determinants of IMT and elasticity. Variables were entered if the respective alpha probability was <0.15 and were removed if it was >0.15. To evaluate whether multiple CVRFs was independently associated with carotid IMT and elasticity, the models were rerun after adding in multiple CVRFs as a dummy variable. To confirm the significance of multiple CVRFs, an additional set of models were constructed that included the individual components of CVRFs (but without multiple CVRFs) as well as all of the possible interactions among these components. To illustrate the contribution of multiple CVRFs to the values of IMT and elasticity, these values were calculated with the least-squares method after adjusting for: 1) age and gender; 2) age, gender, and dyslipidemia; and 3) age, gender, BMI and the individual components of CVRFs. For each adjustment, the values were computed in the absence or presence of multiple CVRFs in the model, and they were compared by ANOVA.

## Results

The prevalence of multiple CVRFs in this study population was 31.8%. Table 1 shows the clinical characteristics of study participants. The values of all the anthropometric, BP and fasting plasma glucose exhibited significant differences in patients with multiple CVRFs than in controls. Patients with multiple CVRFs were, on average, eleven years older (p<0.0001) and more likely to be smoker (41.8% vs. 9.3%, p<0.0001) than controls. IMT was, on average, 43% higher, and elastic modulus was, on average, 60% higher in multiple CVRFs patients than in controls.

**Table 1:** Clinical characteristics of study participants.

Variables	Control subjects (n = 118)	Multiple CVRFs Patients (n = 55)	p Value
Age(yrs)	44.6±14.9	55.6±12.5	0.0001
Female(%)	58.5	43.6	0.071
BMI(kg/m^2^)	22.2±2.65	26.27±3.15	0.0001
Smoker(%)	9.3	41.8	0.0001
SBP(mmHg)	119.3±12.6	134.3±18.2	0.0001
DBP(mmHg)	74.94±8.16	80.0±11.8	0.005
PP(mmHg)	44.36±9.49	54.3±14.1	0.0001
FPG(mmol/L)	6.34±3.65	8.04±2.96	0.001
IMT(mm)	0.554±0.2	0.794±0.309	0.0001
Elastic modulus(kPa)	532±282	851±296	0.0001

Data are presented as the mean value ± SD or percentage of subjects. BMI = body mass index; SBP = systolic blood pressure; DBP = diastolic blood pressure; PP = pulse pressure; FPG = fasting plasma glucose; IMT = intima-media thickness; CVRFs = cardiovascular risk factors.

Table 2 shows the prevalence of multiple CVRFs and its individual components by quartiles of age. The prevalence of hypertension, dyslipidemia, diabetes and multiple CVRFs increased with advancing age group quartiles, whereas the peak prevalence of overweight and smoking appeared in middle age. Furthermore, the value of IMT and elastic modulus increased with advancing age groups.

**Table 2:** Prevalence of individual components and clusters of components of the cardiovascular risk factors by age quartile.

Variables	First Quartile (n = 42)	Second Quartile (n = 43)	Third Quartile (n = 44)	Fourth Quartile (n = 44)	p Value
Age(yrs)	27±4	43±4	55±3	66±5	0.0001
Female(%)	57.1	30.2	65.9	61.4	0.003
Hypertension(%)	0	25.6	31.8	63.6	0.0001
Dyslipidemia(%)	23.8	62.8	56.8	68.2	0.0001
Diabetes(%)	14.3	44.2	52.3	63.6	0.0001
Overweight(%)	16.7	39.5	27.3	29.6	0.138
Smoking(%)	4.8	41.9	13.6	18.2	0.0001
Multiple CVRFs(%)	7.14	37.2	29.6	52.3	0.0001
IMT(mm)	0.399±0.08	0.571±0.16	0.672±0.19	0.869±0.30	0.0001
Elastic modulus(kPa)	272.1±103.6	584.1±178	707.8±249.1	951.5±269.9	0.0001

By ANOVA. Data are presented as the mean value ± SD or percentage of subjects. The abbreviations as in Table 1.

### Effects of Multiple CVRFs on Carotid IMT and Elasticity

Multiple regression models were used to evaluate the independent contributions of multiple CVRFs on carotid IMT and elasticity. A first set of models included age, gender, SBP, DBP, PP and each individual’s component of CVRFs. Age, gender and hypertension were each independently associated with IMT (model R^2^ = 0.473, p<0.0001); age, DBP and PP were each independently associated with elasticity (model R^2^ = 0.696, p<0.0001). When the first set of models was rerun after adding multiple CVRFs as a dummy variable, the variables remained independently associated with IMT and elasticity, respectively, as shown in Table 3. Furthermore, multiple CVRFs was also found to be independently associated with both IMT (p  = 0.042) and elasticity (p  = 0.008), and accounting for 1.3% and 1.2% of the variability in IMT and elasticity, respectively.

**Table 3:** Multiple regression models evaluating the independent determinants of carotid intima-medial thickness and elasticity.

Variables	Multiple CVRFs Not Added to Model		Variables	Multiple CVRFs Added to Model	
	Coefficient	p Value		Coefficient	p Value
Intima-Medial Thickness (mm)					
Age	0.0093	0.0001	Age	0.0091	0.0001
Gender	–0.070	0.018	Gender	–0.059	0.049
Hypertension	0.137	0.0001	Multiple CVRFs	0.078	0.042
			Hypertension	0.099	0.017
Model R^2^	0.473	0.0001	Model R^2^	0.486	0.0001
Elasticity (kPa)					
Age	13.1	0.0001	Age	12.6	0.0001
DBP	5.4	0.0001	DBP	4.6	0.001
PP	8.0	0.0001	Multiple CVRFs	87	0.008
			PP	7.1	0.0001
Model R^2^	0.696	0.0001	Model R^2^	0.708	0.0001

All the models included age, gender, SBP, DBP, PP and each individual component of CVRFs as independent variables. Abbreviations as in Table 1.

A second set of multiple regressions models were constructed for both IMT and elasticity. These models included age, gender, the individual components of CVRFs, and interaction terms representing all of the possible interactions among these components as independent variables. The results indicated that several terms representing the interaction of three or more components of CVRFs were independently associated with carotid IMT and elasticity. This verifies that interactions among the individual components of CVRFs exert synergistic effects on IMT and elasticity. Overall, the model (R^2^ = 0.567 for IMT and R^2^ = 0.774 for elasticity) was modestly increased by the addition of these interaction terms (R^2^ = 0.504 for IMT and R^2^ = 0.732 for elasticity without the interaction terms). It could be due to the dominant effect of age in accounting for the alteration in IMT (40.9%) and elasticity (59.6%).

To assess the contribution of multiple CVRFs to the values of IMT and elastic modulus, the least-squares method was used to calculate these values after adjusting for: 1) age and gender; 2) age, gender, and dyslipidemia; and 3) age, gender, BMI, and the individual components of CVRFs. For each adjustment, the values were calculated in the absence and presence of multiple CVRFs in the model. By ANOVA, the addition of multiple CVRFs to the models significantly increased the values of IMT and elasticity for all three adjustments, as shown in Table 4.

**Table 4:** Mean adjusted values of carotid intima-media thickness and elastic modulus, calculated by the least mean squares method, in the absence or presence of multiple CVRFs.

	Adjusted for Age and Gender	Adjusted for Age, Gender and Dyslipidemia	Multivariate Model
IMT (mm)			
No	0.592±0.018	0.598±0.019	0.577±0.022
Yes	0.713±0.028	0.701±0.03	0.745±0.038
p Value	0.001	0.007	0.001
Elastic modulus (kPa)			
No	587.4±18.24	587.8±18.98	594.2±21.79
Yes	731.7±27.48	730.9±29.61	717.2±37.63
p Value	0.0001	0.0001	0.017

The multivariate model included age, gender, BMI, dyslipidemia, diabetes, overweight and smoking. Data are presented as the mean value ± SD. Abbreviations as in Table 1.

**Figure 1:**
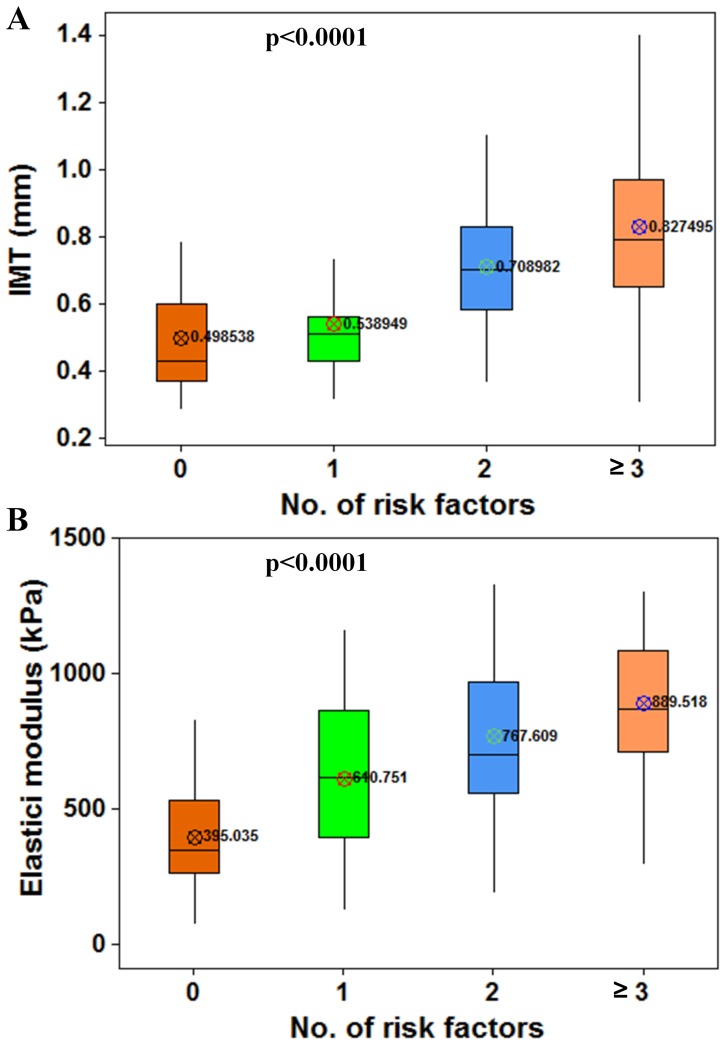
Common carotid intima-media thickness (IMT) (A) and elastic modulus (B) by the number of risk factors.

Subjects were classified as having zero (n  = 47), one (n  = 28), two (n  = 43), or multiple (n  = 55) CVRFs. The carotid IMT and elastic modulus in subjects with different numbers of CVRFs is shown in Figure 1. By ANOVA, adjusted with age as a covariate, the higher the number of CVRFs, the greater IMT and elastic modulus. Compared to subjects without CVRFs, subjects with single, double, and multiple CVRFs, increased by 8.1%, 42.2%, and 66% for IMT, 54.6%, 94.3%, and 125.2% for elastic modulus, respectively.

## Discussion

The major findings of this study are that multiple CVRFs increases carotid IMT and elasticity across all age groups, and multiple CVRFs exerts its effects on carotid structure and function independent of its individual components.

The measurement of carotid IMT has gained acceptance as a noninvasive method to assess the extent of CVD [2], [8], [20]–[22]. Carotid IMT is significantly related to CVRFs and to the extent of CVD. Davis et al. [23] found that higher carotid IMT in 346 men and 379 women aged 33 to 42 years is associated with childhood and current CVRFs. Urbina et al. [24] studied a sample of 518 black and white subjects (mean age 32 years), and found that healthy and asymptomatic young adults with multiple CVRFs displayed increased IMT in the CCA and carotid artery bulb. In studying 1809 subjects (aged 32±5 years), Koskinen et al. [25] found that conventional risk factors and metabolic syndrome are associated with accelerated IMT progression in young adults. In this study, we found that there was an increasing trend in carotid IMT values according to the number of risk factors (Figure 1A), and multiple CVRFs was an independent predictor of carotid IMT (Table 3).

A few previous studies have investigated the relationship between CVRFs and vascular elasticity. In studying 993 subjects at high risk, aged 35–64 years, Jacques et al. [26] found that the cumulative influence of risk factors, even treated, was an independent determinant of arterial stiffness, assessed by carotid-femoral pulse wave velocity measurements. Scuteri et al. [27] found that the clustering of multiple components of the metabolic syndrome is independently associated with increased CCA stiffness, evaluated by the stiffness index. This study verifies these findings (Figure 1B) and extends them insofar as: 1) a different elasticity index - elastic modulus were used, which has been shown to accurately measure arterial elasticity noninvasively; 2) was across a broad age range and showed that the association between multiple CVRFs and carotid elasticity was significant across all age groups (Table 2). Moreover, we found that multiple CVRFs itself was an independent predictor of carotid elasticity (Table 3). Furthermore, carotid elasticity is increasingly recognized as a potent independent predictor of adverse cardiovascular outcomes.

Thus, multiple CVRFs was independently associated with IMT or elasticity, even if several of the individual components of CVRFs were not, suggesting that the clustering of these components interacted to exert synergistic effects on vascular structure and function. This finding supports the concept that multiple CVRFs have a synergistic effect on morbidity and mortality from CVD, which has been demonstrated by epidemiologic studies such as the Framingham Study [28].

### Current Risk Factors

Cardiovascular diseases have a multifactorial etiology. Established traditional risk factors include hypertension, dyslipidemia, diabetes, overweight and smoking [29]. In this study, the CVRFs we evaluated were the traditional risk factors. However, more recent analyses showed that increased triglyceride levels were associated with increased CVD risk [30], [31]. In particular, triglyceride-rich lipoprotein remnants associated with apolipoprotein C-III appeared to have a major impact on risk [32], [33]. As promising novel risk factors, the additive value of homocysteine and high-sensitivity C-reactive protein for assessment of CVD risk was being evaluated in ongoing prospective studies [34]–[36]. Furthermore, genetic information has also been proven as a direct impact on cardiovascular patient care [37]. Therefore, continued focus on newer factors is warranted, as they may further improve the ability to predict future risk and determine treatment when they are included along with the traditional risk factors in the global risk profile.

### Study Limitations

Some limitations of our study should be noted. Firstly, due to the lack of a well-defined physical activity question, we were unable to assess this risk factor in our investigation. Respondents’ smoking status was based on self-report and thus may be subject to reporting bias. Secondly, we measured CCA IMT. Measurements of IMT in the CCA are more reliable and less difficult than IMT measurements in the carotid bifurcation or in the internal carotid artery but also less sensitive to local vascular changes. Therefore, it is possible that the IMT data from only one site may underestimate the relationships between multiple CVRFs and IMT progression compared with using data from all three segments. Furthermore, because our study cohort was racially homogeneous, the generalizability of our findings might be limited to Chinese subjects. Future studies that include more racially and socioeconomically diverse populations are needed to further investigate the relationship between multiple CVRFs and vascular structure and function. In addition, the BP significantly increases from the central to the peripheral arteries [38]. It is possible that brachial BP measurement is an inadequate way to obtain central elasticity, since it overestimates the CCA elastic modulus. To get accurate estimates, central vascular properties must be derived from central measurements. However, there was a fixed difference in central and brachial measurements of PP, the numerator of the elastic modulus, which should lead to a fixed, systematic error in the elastic modulus.

In conclusion, this study indicates that multiple CVRFs is independently associated with increased carotid IMT and elastic modulus, and has a greater impact on carotid IMT and elastic modulus than individual components of CVRFs.

## References

[1] Fagard RH, Pardaens K, Staessen JA, Thijs L (2001) The pulse pressure-to-stroke index ratio predicts cardiovascular events and death in uncomplicated hypertension. J Am Coll Cardiol 38: 227–231. 11451279 10.1016/s0735-1097(01)01362-6

[2] Lorenz MW, Markus HS, Bots ML, Rosvall M, Sitzer M (2007) Prediction of clinical cardiovascular events with carotid intima-media thickness. Circulation 115: 459–467. 17242284 10.1161/CIRCULATIONAHA.106.628875

[3] Stein JH, Inaba Y, Chen JA, Bergmann SR, von Schacky C, et al (2011) Carotid intima–media thickness and cardiovascular events. N Engl J Med 365: 1640–1642. 10.1056/NEJMc1109714 22029989

[4] Manisty C, Mayet J, Tapp RJ, Parker KH, Sever P, et al (2010) Wave reflection predicts cardiovascular events in hypertensive individuals independent of blood pressure and other cardiovascular risk factors. J Am Coll Cardiol 56: 24–30. 20620713 10.1016/j.jacc.2010.03.030

[5] Frimodt-Møller M, Kamper A-L, Strandgaard S, Kreiner S, Nielsen AH (2012) Beneficial effects on arterial stiffness and pulse-wave reflection of combined enalapril and candesartan in chronic kidney disease-a randomized trial. PloS One 7: e41757. 22860014 10.1371/journal.pone.0041757 PMC3409235

[6] Sanz J, Kariisa M, Dellegrottaglie S, Prat-González S, Garcia MJ, et al (2009) Evaluation of pulmonary artery stiffness in pulmonary hypertension with cardiac magnetic resonance. J Am Coll Cardiol 2: 286–295. 10.1016/j.jcmg.2008.08.007 19356573

[7] Magnussen CG, Venn A, Thomson R, Juonala M, Srinivasan SR, et al (2009) The association of pediatric low-and high-density lipoprotein cholesterol dyslipidemia classifications and change in dyslipidemia status with carotid intima-media thickness in adulthood. J Am Coll Cardiol 53: 860–869. 19264243 10.1016/j.jacc.2008.09.061 PMC2759186

[8] Mazzone T, Meyer PM, Feinstein SB, Davidson MH, Kondos GT, et al (2006) Effect of pioglitazone compared with glimepiride on carotid intima-media thickness in type 2 diabetes. JAMA 296: 2572–2581. 17101640 10.1001/jama.296.21.joc60158

[9] Jatoi NA, Jerrard-Dunne P, Feely J, Mahmud A (2007) Impact of smoking and smoking cessation on arterial stiffness and aortic wave reflection in hypertension. Hypertension 49: 981–985. 17372029 10.1161/HYPERTENSIONAHA.107.087338

[10] Weingärtner O, Pinsdorf T, Rogacev KS, Blömer L, Grenner Y, et al (2010) The relationships of markers of cholesterol homeostasis with carotid intima-media thickness. PloS One 5: e13467. 20976107 10.1371/journal.pone.0013467 PMC2956704

[11] Manco M, Bedogni G, Monti L, Morino G, Natali G, et al (2010) Intima-media thickness and liver histology in obese children and adolescents with non-alcoholic fatty liver disease. Atherosclerosis 209: 463–468. 19897197 10.1016/j.atherosclerosis.2009.10.014

[12] Kelly TN, Gu D, Chen J, Huang J, Duan X, et al (2008) Hypertension subtype and risk of cardiovascular disease in Chinese adults. Circulation 118: 1558–1566. 18809800 10.1161/CIRCULATIONAHA.107.723593 PMC2735390

[13] Yang W, Lu J, Weng J, Jia W, Ji L, et al (2010) Prevalence of diabetes among men and women in China. N Engl J Med 362: 1090–1101. 20335585 10.1056/NEJMoa0908292

[14] Lavie CJ, Milani RV, Ventura HO (2009) Obesity and cardiovascular disease: risk factor, paradox, and impact of weight loss. J Am Coll Cardiol 53: 1925–1932. 19460605 10.1016/j.jacc.2008.12.068

[15] Rigotti NA, Pipe AL, Benowitz NL, Arteaga C, Garza D, et al (2010) Efficacy and safety of varenicline for smoking cessation in patients with cardiovascular disease. Circulation 121: 221–229. 20048210 10.1161/CIRCULATIONAHA.109.869008 PMC4096941

[16] Malloy MJ, Kane JP (2012) Hyperlipidemia and cardiovascular disease. Curr Opin Lipidol 23: 591–592. 23160407 10.1097/MOL.0b013e328359f162

[17] Alberti K, Eckel RH, Grundy SM, Zimmet PZ, Cleeman JI, et al (2009) Harmonizing the metabolic syndrome. Circulation 120: 1640–1645. 19805654 10.1161/CIRCULATIONAHA.109.192644

[18] The Expert Panel on Detection E, Treatment of High Blood Cholesterol in Adults (2001) Executive summary of the third report of the National Cholesterol Education Program (NCEP) expert panel on detection, evaluation, and treatment of high blood cholesterol in adults (Adult Treatment Panel III). JAMA 285: 2486–2497. 11368702 10.1001/jama.285.19.2486

[19] Niu L, Qian M, Song R, Meng L, Liu X, et al (2012) A texture matching method considering geometric transformations in noninvasive ultrasonic measurement of arterial elasticity. Ultrasound Med Biol 38: 524–533. 22266234 10.1016/j.ultrasmedbio.2011.12.010

[20] Bots ML, Hoes AW, Koudstaal PJ, Hofman A, Grobbee DE (1997) Common carotid intima-media thickness and risk of stroke and myocardial infarction. Circulation 96: 1432–1437. 9315528 10.1161/01.cir.96.5.1432

[21] Raitakari OT, Juonala M, Kähönen M, Taittonen L, Laitinen T, et al (2003) Cardiovascular risk factors in childhood and carotid artery intima-media thickness in adulthood. JAMA 290: 2277–2283. 14600186 10.1001/jama.290.17.2277

[22] Li Z, Froehlich J, Galis ZS, Lakatta EG (1999) Increased expression of matrix metalloproteinase-2 in the thickened intima of aged rats. Hypertension 33: 116–123. 9931091 10.1161/01.hyp.33.1.116

[23] Davis PH, Dawson JD, Riley WA, Lauer RM (2001) Carotid intimal-medial thickness is related to cardiovascular risk factors measured from childhood through middle age the Muscatine study. Circulation 104: 2815–2819. 11733400 10.1161/hc4601.099486

[24] Urbina EM, Srinivasan SR, Tang R, Bond MG, Kieltyka L, et al (2002) Impact of multiple coronary risk factors on the intima-media thickness of different segments of carotid artery in healthy young adults. Am J Cardiol 90: 953. 12398961 10.1016/s0002-9149(02)02660-7

[25] Koskinen J, Kähönen M, Viikari JSA, Taittonen L, Laitinen T, et al (2009) Conventional cardiovascular risk factors and metabolic syndrome in predicting carotid intima-media thickness progression in young adults. Circulation 120: 229–236. 19581494 10.1161/CIRCULATIONAHA.108.845065

[26] Amar J, Ruidavets JB, Chamontin B, Drouet L, Ferrières J (2001) Arterial stiffness and cardiovascular risk factors in a population-based study. J Hypertens 19: 381–387. 11288807 10.1097/00004872-200103000-00005

[27] Scuteri A, Najjar SS, Muller DC, Andres R, Hougaku H, et al (2004) Metabolic syndrome amplifies the age-associated increases in vascular thickness and stiffness. J Am Coll Cardiol 43: 1388–1395. 15093872 10.1016/j.jacc.2003.10.061

[28] Kannel WB (1988) Contributions of the Framingham study to the conquest of coronary artery disease. Am J Cardiol 62: 1109–1112. 3189175 10.1016/0002-9149(88)90558-9

[29] The Expert Panel (2002) Expert panel on detection, evaluation, and treatment of high blood cholesterol in adults final report. Circulation 106: 3143–3421. 12485966

[30] Assmann G, Schulte H, von Eckardstein A (1996) Hypertriglyceridemia and elevated lipoprotein (a) are risk factors for major coronary events in middle-aged men. Am J Cardiol 77: 1179. 8651092 10.1016/s0002-9149(96)00159-2

[31] Yarnell J, Patterson C, Sweetnam P, Thomas H, Bainton D, et al (2001) Do total and high density lipoprotein cholesterol and triglycerides act independently in the prediction of ischemic heart disease?. Arterioscler Thromb Vasc Biol 21: 1340–1345. 11498463 10.1161/hq0801.093505

[32] Luc G, Fievet C, Arveiler D, Evans A, Bard JM, et al (1996) Apolipoproteins C-III and E in apoB-and non-apoB-containing lipoproteins in two populations at contrasting risk for myocardial infarction. J Lipid Res 37: 508–517. 8728314

[33] Ooi E, Barrett P, Chan D, Watts G (2008) Apolipoprotein C-III: understanding an emerging cardiovascular risk factor. Clin Sci 114: 611–624. 18399797 10.1042/CS20070308

[34] Ridker PM (2001) High-sensitivity C-reactive protein: potential adjunct for global risk assessment in the primary prevention of cardiovascular disease. Circulation 103: 1813–1818. 11282915 10.1161/01.cir.103.13.1813

[35] Clarke R, Bennett DA, Parish S, Verhoef P, Dötsch-Klerk M, et al (2012) Homocysteine and coronary heart disease: Meta-analysis of MTHFR case-control studies, avoiding publication bias. PLoS Med 9: e1001177. 22363213 10.1371/journal.pmed.1001177 PMC3283559

[36] Elliott P, Chambers JC, Zhang W, Clarke R, Hopewell JC, et al (2009) Genetic loci associated with C-reactive protein levels and risk of coronary heart disease. JAMA 302: 37–48. 19567438 10.1001/jama.2009.954 PMC2803020

[37] Ginsburg GS, Shah SH, McCarthy JJ (2007) Taking cardiovascular genetic association studies to the next level. J Am Coll Cardiol 50: 930–932. 17765118 10.1016/j.jacc.2007.05.025

[38] O'ROURKE MF, Blazek JV, Morreels CL, Krovetz LJ (1968) Pressure wave transmission along the human aorta. Circ Res 23: 567–579. 5677947 10.1161/01.res.23.4.567

